# Efficient differentiation of *Nocardia farcinica*, *Nocardia cyriacigeorgica* and *Nocardia beijingensis* by high-resolution melting analysis using a novel locus

**DOI:** 10.1099/jmm.0.001205

**Published:** 2020-06-01

**Authors:** Shuai Xu, Xuexin Hou, Dan Li, Lina Sun, Minghui Li, Xingzhao Ji, Xuebing Wang, Zhenjun Li

**Affiliations:** ^1^​ State Key Laboratory for Infectious Disease Prevention and Control, National Institute for Communicable Disease Control and Prevention, Chinese Center for Disease Control and Prevention, Beijing, PR China

**Keywords:** bacterial identification, *fusA-tuf *IR locus, high-resolution melting analysis, *Nocardia *species

## Abstract

Accurate identification of *Nocardia* species remains a challenge due to the complexities of taxonomy and insufficient discriminatory power of traditional techniques. We report the development of a molecular technique that utilizes real-time PCR-based high-resolution melting (HRM) analysis for differentiation of the most common *Nocardia* species. Based on a novel *fusA-tuf* intergenic region sequence, *Nocardia farcinica *, *Nocardia cyriacigeorgica* and *Nocardia beijingensis* were clearly distinguished from one another by HRM analysis. The limit of detection of the HRM assay for purified *Nocardia* spp. DNA was at least 10 fg. No false positives were observed for specificity testing of 20 non-target clinical samples. In comparison to established matrix-assisted laser desorption/ionization-time of flight MS, the HRM assay improved the identification of *N. beijingensis *. Additionally, all the products of PCR were verified by direct sequencing. In conclusion, the developed molecular assay allows simultaneous detection and differentiation of *N. farcinica *, *N. cyriacigeorgica* and *N. beijingensis* with high sensitivity and specificity.

*Nocardia* spp., which are ubiquitous in the environment, are long-neglected opportunistic pathogens, with the majority of infections occurring in immunocompromised and immunodeficient patients [1, 2]. *Nocardia farcinica* and *Nocardia cyriacigeorgica* are the most common pathogens causing nocardiosis in various parts of the world [3–5]. *Nocardia beijingensis* is frequently isolated in China, Thailand and Japan; however, infections have also been observed outside of Asia in recent years [6–8]. The main clinical symptom of nocardiosis consists of lung abscesses, but other symptoms include brain abscesses, skin abscesses and even life-threating systemic disseminated diseases [9–12]. These diverse species have different pathogenic characteristics and drug-sensitivity patterns, and cause pulmonary disease phenotypes that are easily confused with diseases caused by other bacteria or fungi [13, 14]. In many cases, patients with nocardiosis are misdiagnosed and/or mistreated, resulting in high hospital mortality [15]. With the increasing numbers of AIDS patients and organ-transplant patients, and the widespread use of clinical immunosuppressants, the occurrence of *Nocardia* infections has increased [16, 17]. Hence, there is an urgent need to develop a simple, rapid, sensitive and specific diagnostic technique for the detection of *Nocardia *.

Traditionally, the identification of *Nocardia* spp. has been based on microscopic morphology and biochemical reactions, which remain challenging [18]. Sequence polymorphisms within 16S rRNA, *hsp65*, *gyrB* and *rpoB* have been evaluation for the identification of *Nocardia* [9, 19–21]. However, these loci are too conserved to differentiate some closely related species [22]. Identification via matrix-assisted laser desorption/ionization-time of flight (MALDI-TOF) MS requires access to expensive laboratory instruments and specialized personnel, and some difficult-to-identify *Nocardia* species might still require additional molecular tests for accurate identification [23, 24].

High-resolution melting (HRM) analysis is a method of genetic analysis that can detect single-nucleotide polymorphisms [25]. HRM assays characterize amplified PCR products according to their dissociation behaviour without the need for further separation steps, such as gel electrophoresis. A fluorescently labelled dye that can be inserted into dsDNA is combined with amplicons produced from the PCR. As the temperature increases, the dsDNA dissociates into single strands, leading to decreases in fluorescent intensity [26]. The melting temperature (*T*_m_) depends on amplicon length, the GC/AT ratio and nucleotide sequence. Different melting curves can be formed according to fluorescent intensity and the *T*_m_ value. The HRM assay has already been used successfully to identify many important viruses, fungi and bacteria [27–29]. In the present study, a simple, rapid and cost-effective technique using real-time PCR coupled with the HRM assay was developed for identification of *N. farcinica *, *N. cyriacigeorgica* and *N. beijingensis* at the species level using a novel *fusA-tuf* intergenic region (IR) sequence.

Reference strains *N. farcinica* IFM 10152, *N. cyriacigeorgica* DSM 44484 and *N. beijingensis* DSM 44636 used in this study were obtained from the DSMZ (Leibniz-Institut DSMZ – Deutsche Sammlung von Mikroorganismen und Zellkulturen, Brunswick, Germany). In addition to the reference strains, 85 clinical isolates were collected from 15 provinces in China between 2013 and 2018. Isolates were identified by DNA sequencing of 16S rRNA genes, as described elsewhere [30]. Briefly, the complete 16S rRNA genes of all clinical isolates were sequenced. Each 16S rRNA was compared with those available in GenBank using the blastn program, with species-level calls made by ≥99 % identity.

The *fusA* and *tuf* genes, encoding elongation factors G and Tu, respectively, are highly conserved genes among bacteria [31]. The location where these two genes are separated by a short (<120 bp) non-conserved region may give more discriminatory power. Thus, the region that covers the last 149 bp of *fusA*, the first 56 bp of *tuf* and the IR between them – designated as *fusA-tuf* IR – was selected for further analysis.

The sequences of the *fusA-tuf* IR locus for three reference strains (* N. farcinica* IFM 10152, *N. cyriacigeorgica* DSM 44484 and *N. beijingensis* DSM 44636) were obtained from the published genomes in the National Center for Biotechnology Information databases and subjected to a multiple-sequence alignment using SeqMan in the DNAStar software (Fig. 1). A primer pair (forward 5′-TGGTTCCGCTCTCGGAGATG-3′ and reverse 5′-CCGATGGTGCCGATGTTGAC-3′) was designed targeting the conserved region of *fusA-tuf* IR using CmSuite software (v 8.0). The PCR primers amplified a 344, 321 and 303 bp fragments for *N. farcinica *, *N. cyriacigeorgica* and *N. beijingensis *, respectively.

**Fig. 1.**
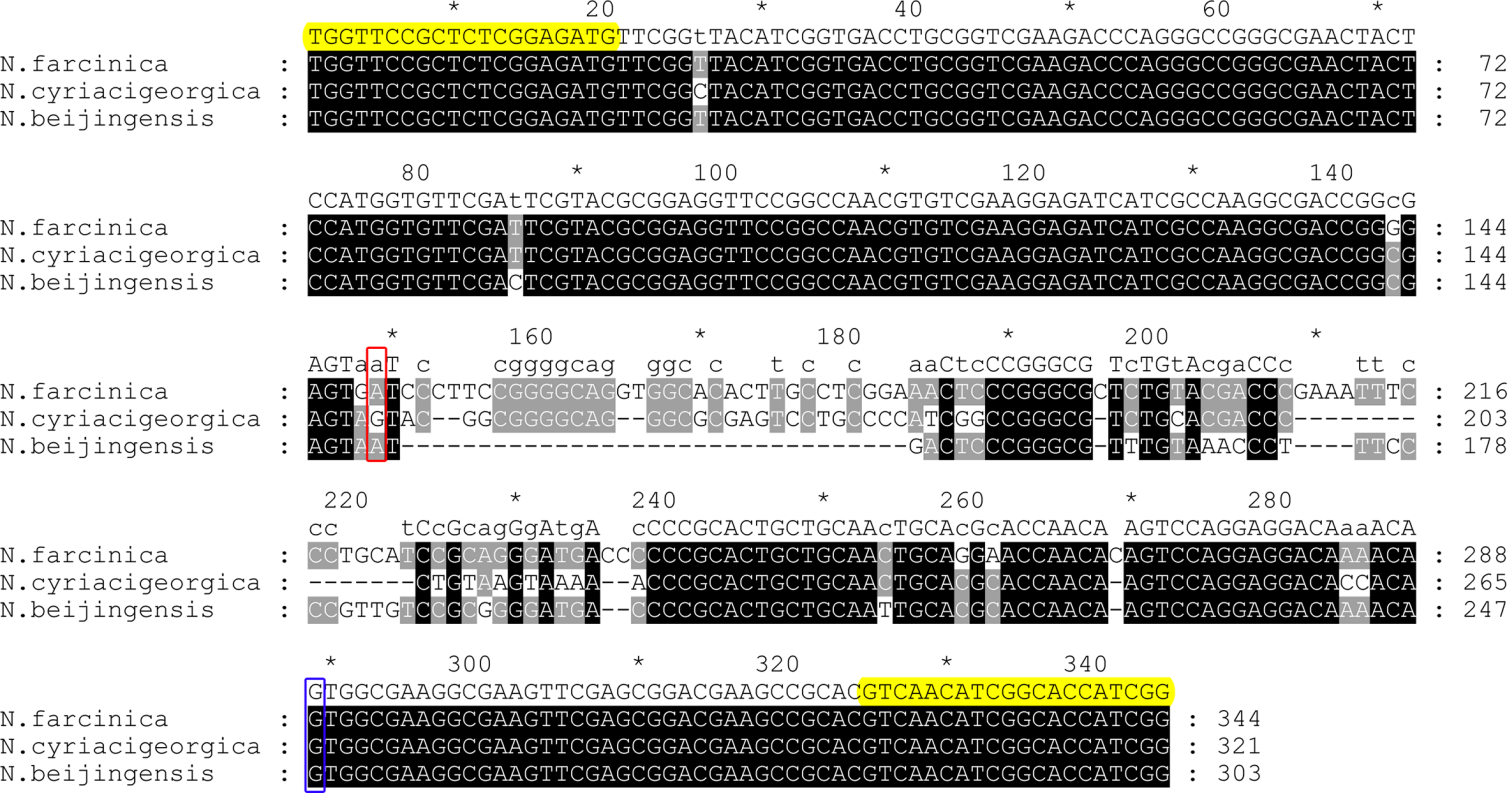
Sequence alignment of the *fusA-tuf* IR locus from each *Nocardia* species. Primer regions are indicated in yellow. In the consensus line, capital letters represent conserved bases, whereas small letters represent substitutions or deletions. Gaps are shown as hyphens. The end of *fusA* and start of *tuf* genes are indicated in the red box and purple box, respectively.

The genomic DNA of reference strains and clinical isolates was extracted using a QIAamp DNA minikit (Qiagen), in accordance with the manufacturer’s instructions. The DNA samples were quantified using a spectrophotometer (NanoDrop ND-1000; Thermo Fisher Scientific) and were kept at −20 °C until further use.

HRM analysis was performed and validated using an ABI QuantStudio 6 Flex system (Thermo Fisher Scientific). Each reaction was set up in a 30 µl volume containing 15 µl 2×TaqMan PCR master mix (Thermo Fisher Scientific), 0.9 µl primer F (10 μΜ), 0.9 μl primer R (10 µM), 0.3 µl ROX reference dye II (100×), 1.5 µl EvaGreen 20× in water (Biotium), 1 µl DNA sample (10 ng µl^−1^) and 10.4 µl ddH_2_O. The amplification protocol consisted of a pre-denaturation at 95 °C for 10 min; followed by 35 cycles of denaturation at 95 °C for 15 s, annealing at 63 °C for 30 s and extension at 72 °C for 30 s. Next, HRM analysis was initiated by raising the temperature to 95 °C for 15 s and decreasing it to 60 °C for 1 min. Then, the melting curves were generated by ramping from 60 to 95 °C at increments of 0.025 °C s^−1^. All of the amplicons were tested in triplicate to detect technical errors. The HRM data were analysed using QuantStudio real-time PCR software v1.3. The experiments were performed in triplicate.

In the HRM analysis, *N. farcinica *, *N. cyriacigeorgica* and *N. beijingensis* each showed a single peak (Fig. 2a). *N. farcinica* had a peak at around 90.0 °C, *N. cyriacigeorgica* had a peak at 91.0 °C and *N. beijingensis* produced a peak at approximately 89.3 °C. The difference plots obtained allowed for clear differentiation of species (Fig. 2b).

**Fig. 2.**
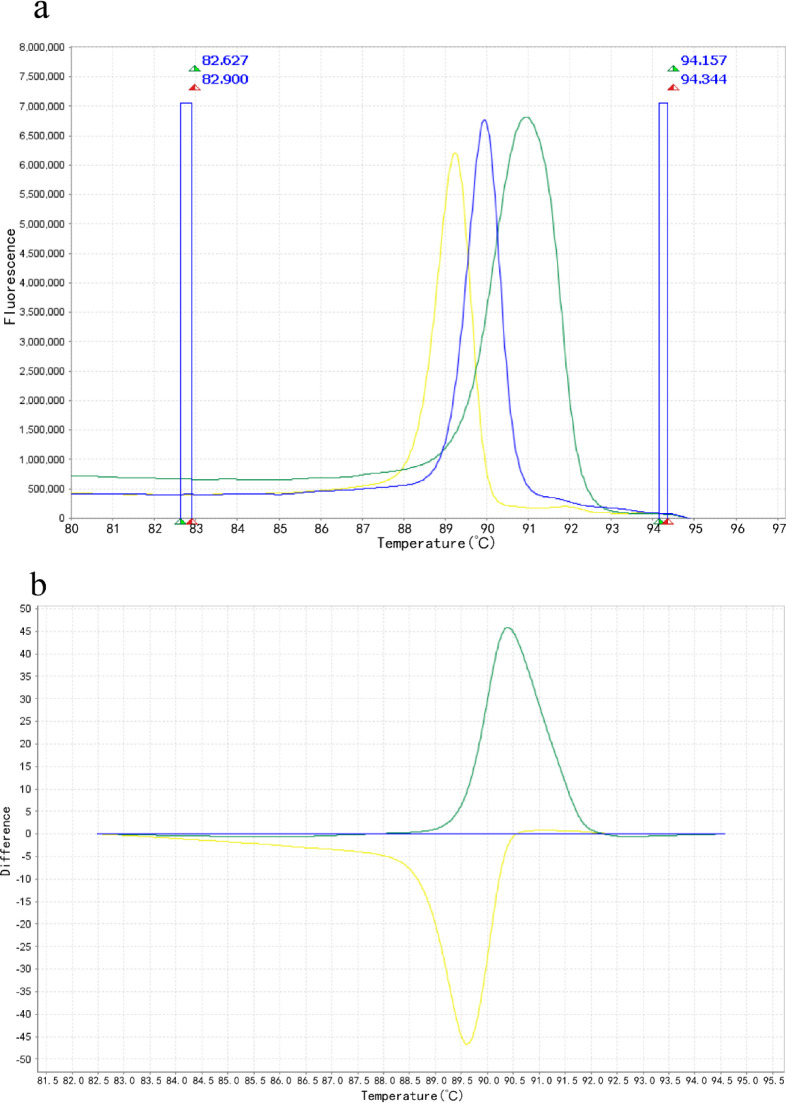
Representative HRM analysis for differentiating *Nocardia* species. Curves of tested samples identified as *N. farcinica* are shown in blue, *N. cyriacigeorgica* in green and *N. beijingensis* in yellow. (a) Melting curves for *N. farcinica *, *N. cyriacigeorgica* and *N. beijingensis *. The blue boxes on the left and right represent pre-melting (82.627–82.900 °C) and post-melting (94.157–94.344 °C) regions, respectively. (b) A difference plot for *N. farcinica *, *N. cyriacigeorgica* and *N. beijingensis *.

To determine the limit of detection, we performed 10-fold serial dilutions of the genomic DNA from three *Nocardia* species (*N. farcinica, N. cyriacigeorgica* and *N. beijingensis *) using sterile ddH_2_O as follows: 10 ng µl^−1^, 100 pg µl^−1^, 10 pg µl^−1^, 1 pg µl^−1^, 100 fg μl^−1^ and 10 fg μl^−1^. HRM reactions were performed with these serial dilutions being used as templates. Sterile ddH_2_O served as a blank control. The experiments were performed in triplicate. Results indicate that the limit of detection for *N. farcinica *, *N. cyriacigeorgica* and *N. beijingensis* was at least 10 fg (Fig. 3).

**Fig. 3.**
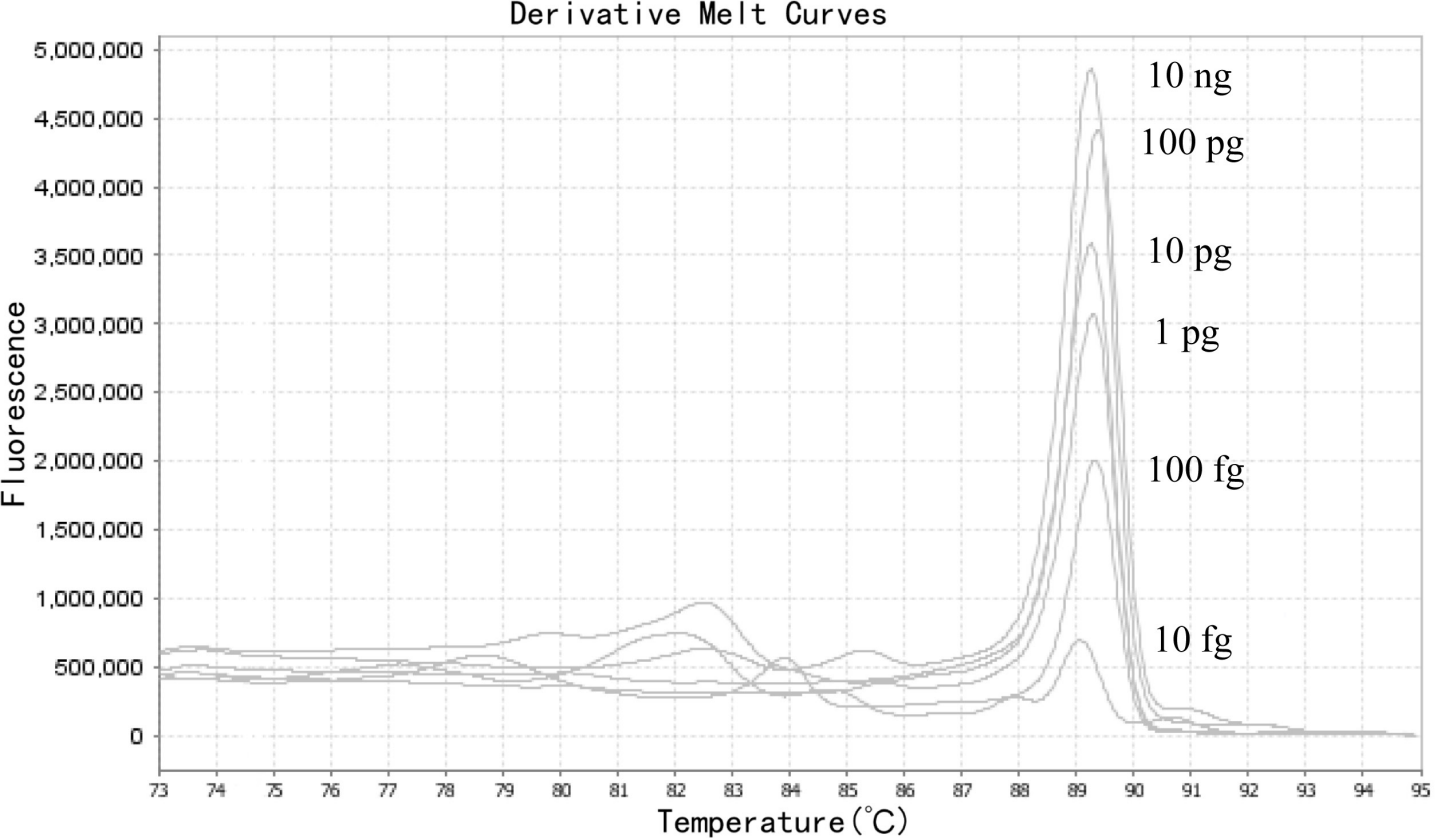
Limit of detection of the HRM assay.

Using the HRM assay, 10 ng genomic DNA of 85 clinical isolates were identified as 58 strains of *N. farcinica *, 23 strains of *N. cyriacigeorgica* and 4 strains of *N. beijingensis *, which is consistent with the 16S rRNA identification method (Fig. S1, available with the online version of this article). The assay’s specificity was also assessed by testing 20 non-* Nocardia* pathogens. These non-target samples included the following: *Corynebacterium diphtheriae *, *Corynebacterium striatum *, *Corynebacterium simulans *, *Mycoplasma pneumoniae *, *Skermania piniformis *, *Arcanobacterium pyogenes *, *Staphylococcus aureus *, *Streptococcus pneumoniae *, *Stenotrophomonas maltophilia *, *Staphylococcus haemolyticus *, *Legionella pneumophila *, *Klebsiella pneumoniae *, *Haemophilus influenzae *, *Neisseria meningitidis *, *Pseudomonas aeruginosa *, a cryptococcal meningitis isolate, *Actinobaculum suis *, *Candida albicans*, a *Pneumocystis jirovecii* pneumonia isolate and *Trichosporon*. Except for *Skermania piniformis *, which produced a melting curve at 83.4 °C, the pathogens showed no melting curves.

To further evaluate the performance of the assay for identification of *Nocardia* species in clinical isolates, a total of 85 isolates were tested in comparison with the established MALDI-TOF MS method, using a Microflex LT mass spectrometer (Bruker Daltonics). The software used for the data acquisition was FlexControl 3.0 (Bruker Daltonics). The parameters used were as follows: mass spectra within 2–20 kDa; ion source 1, 20 kV; ion source 2, 18.5 kV; lens, 6.0 kV; and linear detector, 2560 V. *Escherichia coli* ATCC 8739 was used for mass calibration.

Samples were prepared as described elsewhere [23]. Briefly, single isolated colonies were spotted onto a polished-steel MALDI target plate. Samples were overlaid with 1 µl 70 % formic acid and were allowed to dry. Each dried spot was then overlaid with 1 µl matrix solution (α-cyano-4-hydroxycinnamic acid). All isolates were analysed in duplicate. Species-level identification was accepted if the score values were ≥2.00; genus-level identification was accepted if the score was 1.70–2.00 [32]. The experiments were performed in triplicate.

The results showed that MALDI-TOF MS identified 80 out of 85 (94.1 %) of *Nocardia* isolates to the species level. Among the five remaining strains, two *N. cyriacigeorgica* were assigned to *N. farcinica */* N. cyriacigeorgica *, while three *N. beijingensis* were identified to the genus level. With the application of HRM, 100 % (85) of *Nocardia* isolates were accurately identified. HRM showed a higher resolution to differentiate *N. beijingensis* isolates, indicating our method as a valid alternative to the MALDI-TOF MS assay.

In order to further confirm the reliability and specificity of the HRM assay, nucleotide sequence analysis was performed. After HRM analysis, the PCR products of the 85 clinical isolates used in this study were purified with a QIAquick PCR purification kit (Qiagen), according to the manufacturer’s instructions. Sequencing was performed with the same primers as for HRM using an ABI PRISM 7500 sequence detection system (Applied Biosystems), according to the standard protocol of the supplier. All the sequences of the PCR products were identical to the reference samples, confirming the results of the HRM analysis.

To our knowledge, this HRM assay is the first assay to specifically identify the main species of *Nocardia* by targeting the *fusA-tuf* IR locus. This novel locus used in our study, which combines two adjacent protein-encoding genes, took advantage of the non-homologous region between them; thus, giving more discriminatory power. Based on the *fusA-tuf* IR locus sequence, *N. farcinica *, *N. cyriacigeorgica* and *N. beijingensis* were clearly and reliably distinguished from one another by unique HRM graphs.

Although the HRM assay showed high discriminatory power, some aspects may affect this technology. First, proper primer design is essential for the development of the HRM assay. Improvement of the specificity of the primers and avoidance of the formation of primer dimers is necessary [33]. Second, the length of targeted amplicons should not exceed 400 bp to ensure a good sensitivity of species detection [34]. Moreover, since the MgCl_2_ concentration strongly influences the melting behaviour of dsDNA, it should be optimized carefully [33].

One limitation in this study was that the designed assay was not evaluated for detection of *Nocardia* spp. in human clinical samples, such as sputum. The application of the HRM assay to clinical specimens would make the technique accessible to more laboratories, but we were unable to perform this analysis. As nocardiosis is neglected in the clinic, it was difficult to obtain clinical samples and the informed consents of patients.

However, despite this limitation, our study has major advantages. (i) The three *Nocardia* species causing nocardiosis can be identified using a single primer pair, in a one-step closed-tube system. (ii) Although reverse transcription-PCR holds great utility, it requires fluorescently labelled probes, which results in a substantial increase in cost. In contrast, the HRM assay uses common and widely available reagents and equipment, along with inexpensive unlabelled oligonucleotides. (iii) After the HRM analysis, samples can be discriminated into groups based on the unique melting profiles and limiting the cost of sequencing to only some representative samples of each group.

In conclusion, an HRM method for simultaneous detection of *N. farcinica *, *N. cyriacigeorgica* and *N. beijingensis* based on the *fusA-tuf* IR locus was successfully developed and evaluated. This method represents a rapid, simple and accurate technique to identify *Nocardia* spp. of clinical interest, and could be used as a potential screening tool for *Nocardia* strains in basic and clinical laboratories.

## Supplementary Data

Supplementary material 1
