# Supplementary material for: Efficient differentiation of Nocardia farcinica, Nocardia cyriacigeorgica and Nocardia beijingensis by high-resolution melting analysis using a novel locus
Source: J Med Microbiol. 2020 Jun 1;69(12):1367–72. doi: 10.1099/jmm.0.001205 (PMC7819362; doi:10.1099/jmm.0.001205)
Supplement: Supplementary material 1 [file jmm-69-1367-s001.pdf]

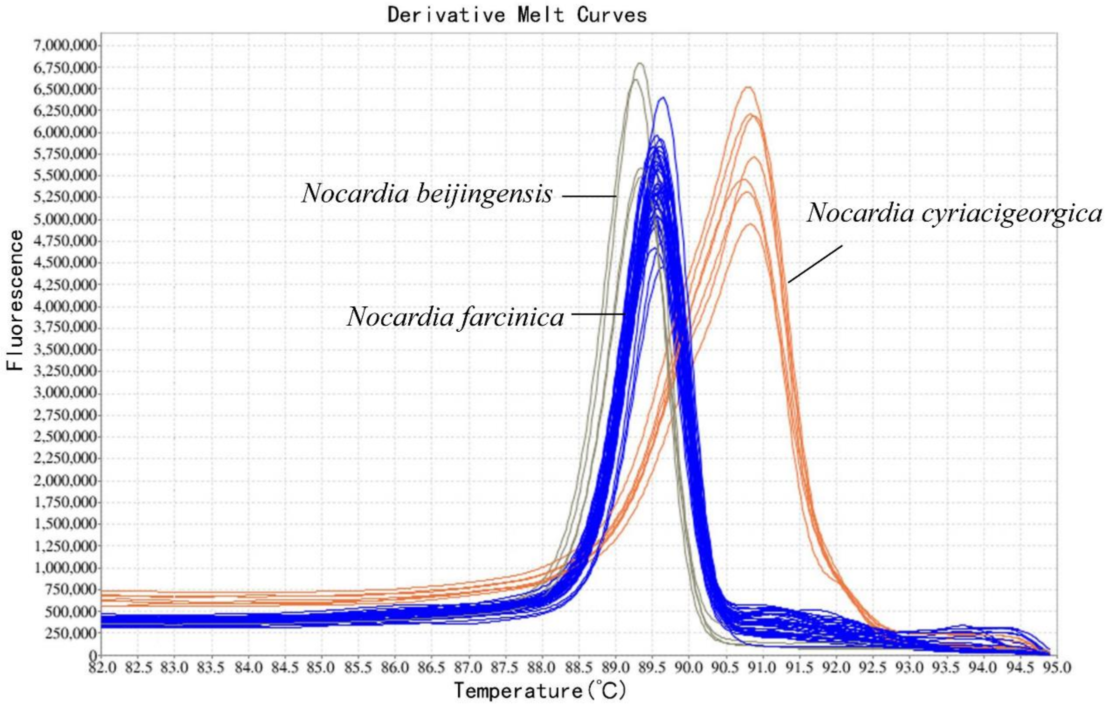

Supplementary Fig. 1. Representative results of clinical isolates tested by High resolution melting graphs.
